# Embryo transfer impact: a comprehensive national cohort analysis comparing maternal and neonatal outcomes across varied embryo stages in fresh and frozen transfers

**DOI:** 10.3389/fendo.2024.1400255

**Published:** 2024-06-12

**Authors:** Chih-Ting Chang, Shih-Feng Weng, Hui-Yu Chuang, I-Le Hsu, Chia-Yi Hsu, Eing-Mei Tsai

**Affiliations:** ^1^ Department of Obstetrics and Gynecology, Kaohsiung Medical University Chung-Ho Memorial Hospital, Kaohsiung, Taiwan; ^2^ Department of Healthcare Administration and Medical Informatics, Kaohsiung Medical University, Kaohsiung, Taiwan; ^3^ Center for Medical Informatics and Statistics, Office of Research and Development, Kaohsiung Medical University, Kaohsiung, Taiwan; ^4^ Center for Big Data Research, Kaohsiung Medical University, Kaohsiung, Taiwan; ^5^ Department of Obstetrics and Gynecology, Kaohsiung Municipal Ta-Tung Hospital, Kaohsiung, Taiwan; ^6^ Graduate Institute of Medicine, College of Medicine, Kaohsiung Medical University, Kaohsiung, Taiwan

**Keywords:** frozen embryo transfer, IVF, blastocyst stage, cleavage stage, assisted reproductive technology

## Abstract

**Introduction:**

The utilization of frozen embryo transfer not only enhances reproductive outcomes by elevating the likelihood of live birth and clinical pregnancy but also improves safety by mitigating the risks associated with ovarian hyperstimulation syndrome (OHSS) and multiple pregnancies. There has been an increasing debate in recent years regarding the advisability of making elective frozen embryo transfer the standard practice. Our study aims to determine the optimal choice between fresh and frozen embryo transfer, as well as whether the transfer should occur at the cleavage or blastocyst stage.

**Method:**

In this retrospective cohort study conducted in Taiwan, data from the national assisted reproductive technology (ART) database spanning from January 1st, 2013, to December 31st, 2017, were analyzed. The study included 51,762 eligible female participants who underwent ART and embryo transfer. Pregnancy outcomes, maternal complications, and singleton neonatal outcomes were evaluated using the National Health Insurance Database from January 1st, 2013, to December 31st, 2018. Cases were categorized into groups based on whether they underwent fresh or frozen embryo transfers, with further subdivision into cleavage stage and blastocyst stage transfers. Exposure variables encompassed clinical pregnancy rate, live birth rate, OHSS, pregnancy-induced hypertension, gestational diabetes mellitus (DM), placenta previa, placental abruption, preterm premature rupture of membranes (PPROM), gestational age, newborn body weight, and route of delivery.

**Results:**

Frozen blastocyst transfers showed higher rates of clinical pregnancy (CPR) and live births (LBR) compared to fresh blastocyst transfers. Conversely, frozen cleavage stage transfers demonstrated lower rates of clinical pregnancy and live birth compared to fresh cleavage stage transfers. Frozen embryo transfers were associated with reduced risks of OHSS but were linked to a higher risk of pregnancy-induced hypertension compared to fresh embryo transfers. Additionally, frozen embryo transfers were associated with a higher incidence of large for gestational age infants and a lower incidence of small for gestational age infants.

**Conclusion:**

The freeze-all strategy may not be suitable for universal application. When embryos can develop to the blastocyst stage, FET is a favorable choice, but embryos can only develop to the cleavage stage, fresh embryo transfer becomes a more reasonable option.

## Introduction

In 1999, the first randomized controlled trial (RCT) comparing frozen embryo transfer (FET) and fresh embryo transfer (ET) was published by Ferraretti et al (1). The results suggested that FET reduced the incidence of ovarian hyperstimulation syndromes (OHSS) and provided comparable pregnancy rates and live birth rates (LBRs) compared to fresh ET. Since then, advancements in vitrification protocols have significantly contributed to the improvement of FET cycles. The conclusion drawn from the systematic reviews in 2013 and in 2019 both written by Matheus Roque et al (2, 3) supports the use of elective FET as a preferred approach in *in vitro* fertilization (IVF)/intracytoplasmic Sperm Injection (ICSI) cycles. This approach not only improves reproductive outcomes by increasing the chances of live birth and clinical pregnancy but also enhances safety by reducing the risks of OHSS and multiple pregnancies. Due to these consistent findings, there has been a growing discussion in recent years about whether it is advisable to adopt elective frozen embryo transfer as the standard practice (4–6). The question “Is frozen embryo transfer the future?” has indeed become a hotly debated topic (7). The need for additional validation arises in the ongoing discussions about potentially establishing elective frozen embryo transfer as the standard practice.

The embryo stage at transfer or the moment of cryopreservation has not always been taken into account even though *in vitro* culture is known to affect perinatal outcomes (8). In comparing the embryo stage at transfer, it was found that blastocyst transfer has higher live birth rate (LBR) and clinical pregnancy rate (CPR) (9–11). In 2016, a RCT conducted in China focused on a cohort of patients diagnosed with polycystic ovary syndrome (PCOS) and sought to compare the outcomes of utilizing cleavage stage embryos in both frozen embryo transfer and fresh embryo transfer procedures (12). The research unveiled that the LBRs were notably higher within the FET group in comparison to the fresh ET group. Two years later, the same research team embarked on a parallel investigation, involving ovulatory women who are considered normal responders to ovarian stimulation (13). Once again, the study analyzed the utilization of cleavage stage embryos, coinciding with the publication of a study from Vietnam within the same year, targeting a non-PCOS population, intriguingly produced similar result (14). In both cases, when the focus shifted to normal responders, the previously observed advantage of FET over fresh ET seemed to diminish, with both groups displaying comparable outcomes in terms of ongoing pregnancy rates (OPR) and LBRs.A relevant question arises regarding the outcomes related to blastocyst stage embryos. In 2017, a British RCT and, subsequently, a multicenter RCT conducted in China in 2019 delved into the utilization of blastocysts in FET as opposed to fresh ET (15, 16). The British study went a step further by incorporating preimplantation genetic testing (PGT), selecting exclusively euploid embryos for transfer. Remarkably, both studies arrived at a consistent conclusion: FET demonstrated superior outcomes in terms of OPR and LBR when compared to fresh ET.

So far, there has not been a cross comparison conducted for blastocyst stage, cleavage stage, frozen, and fresh embryo transfers. Such a comprehensive comparison requires more specific data to determine the differences in reproductive outcomes, complications, and other important outcomes for each method. Therefore, this study aims to compare the outcomes of fresh blastocyst, fresh cleavage stage, frozen blastocyst, and frozen cleavage stage embryo transfers. The findings illuminate the factors that impact live birth rates, maternal complications, and neonatal outcomes. The study will employ a cohort design by analyzing data from Taiwanese national assisted reproduction technology (ART) data set and national population registry data set.

## Materials and methods

This retrospective cohort study was conducted in Taiwan and approved by the institutional review board of Kaohsiung Medical University Chung-Ho Memorial Hospital, IRB-No. KMUHIRB-E(I)-20210222, which waived the requirement for informed consent because the data were encrypted and deidentified.

Couples who entered the IVF treatment in Taiwan have been completely recorded in the Taiwan national ART database. The ART database in Taiwan was established in the year 1998. It collects case data of individuals who undergo artificial reproduction procedures at the respective reproductive institutions, excluding artificial insemination between spouses. This database has undergone de-identification processes, including the removal of directly identifiable fields such as names and addresses. Sensitive fields such as identification numbers, institution codes, insurance policy unit codes, tax identification numbers, dates of birth, medical dates, and admission dates have been masked to comply with the strong data protection standards of FIPS 140–2 Level 3 international security standards. The related data can only be used within the independent operating area set up by the authority, and any disclosed statistical results are carefully reviewed to ensure that there is no possibility of identifying specific individuals through the data application or disclosure methods.

### Participants flow chart

We utilized the national ART dataset to examine the demographic profiles of patients who underwent ART and embryo transfers between January 1, 2013, and December 31, 2017. Our study encompassed all embryo transfer cycles, with the exclusion of cases involving no embryo transfer or duplicate transfers.

Initially, we classified the cases into two distinct groups: frozen embryo transfer and fresh embryo transfer. Subsequently, we further refined the dataset to account for cases with missing embryo implantation data, those with Day 4 embryo transfers, or instances of duplicate implantation days. Within these defined categories, we differentiated between fresh cleavage stage, fresh blastocyst stage, frozen cleavage stage, and frozen blastocyst stage transfers.

In order to ensure the homogeneity and consistency of our dataset, we also excluded cases involving donated oocytes or sperm, as well as couples with unknown causes of infertility or unknown paternal age. Additionally, we excluded cases where alternative assisted reproductive methods, such as gamete intrafallopian transfer (GIFT), zygote intrafallopian transfer/tubal embryo transfer (ZIFT/TET), or preimplantation genetic screening (PGS), were employed. The distribution of individuals within each subgroup was as follows: 18792 cases for fresh cleavage embryo transfer, 5525 for fresh blastocyst transfer, 9549 for frozen cleavage embryo transfer, and 17896 for frozen blastocyst embryo transfer.

**Figure 1:**
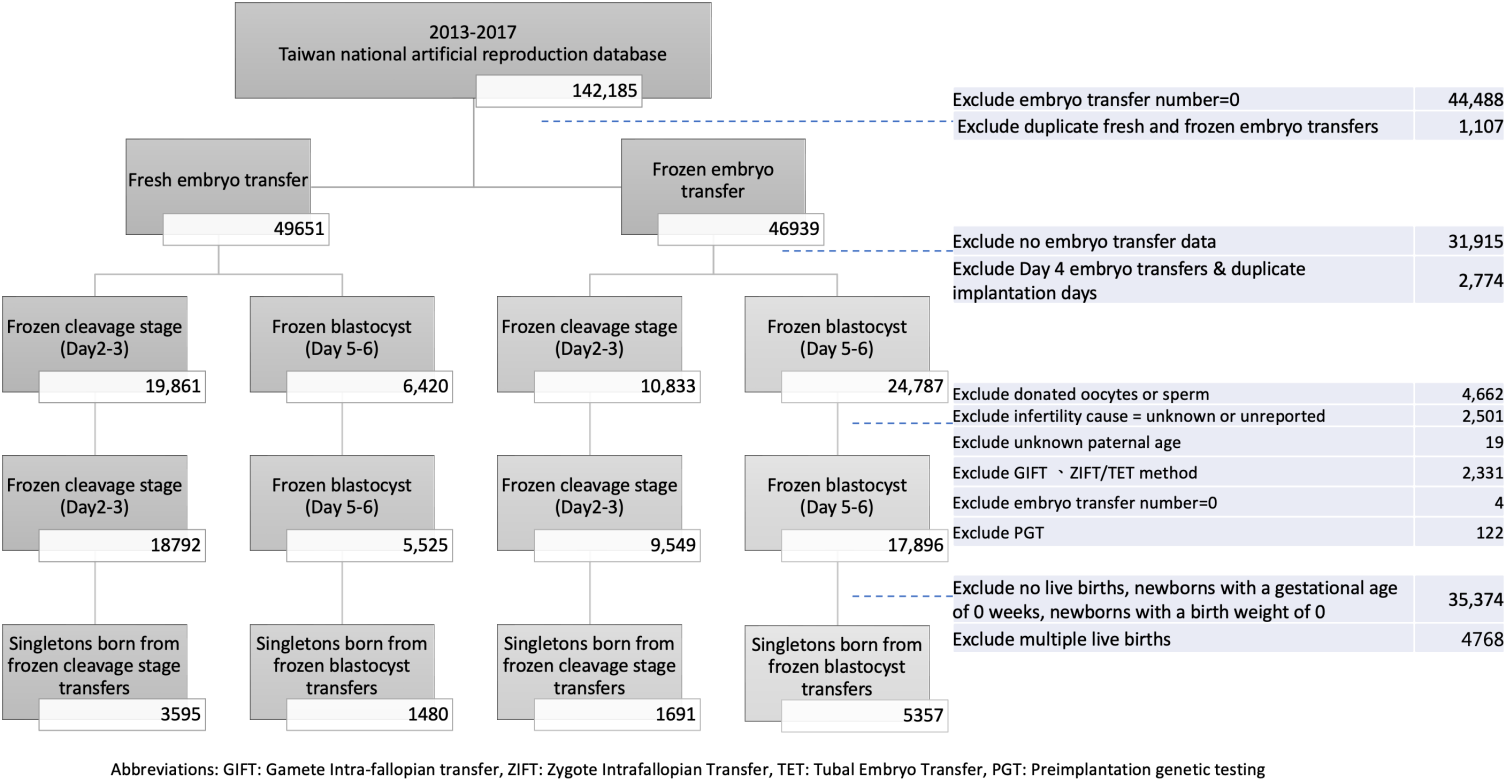
Participant flow chart. GIFT, Gamete intra-fallopian transfer; ZIFT, Zygote Intrafallopian Transfer; TET, Tubal Embryo Transfer; PGT, Preomplantation genetic testing.

Subsequently, we focused on singletons born in all four groups. To ensure data integrity and relevance, cases without recorded instances of live births, newborns with a gestational age of 0 weeks, infants with a birth weight of 0, or cases with live birth greater than one were eliminated from the dataset. The number of singletons in each group was as follows: 3595 for fresh cleavage stage transfers, 1480 for fresh blastocyst transfers, 1691 for frozen cleavage stage transfers, and 5357 for frozen blastocyst transfers (**Figure 1**).

### Study outcomes

Our study outcomes include: (1) Reproductive outcomes: This includes biochemical pregnancies (early pregnancy with an increase in β-hCG [human chorionic gonadotropin] levels), clinical pregnancies (confirmed through ultrasound visualization of the gestational sac), ultrasonographic confirmation of fetal heartbeat, and live births. (2) Maternal complications: Maternal complications were obtained from the National Health Insurance Database and include the following conditions identified by specific codes: pregnancy-induced hypertension (ICD-9 code 642 or ICD-10 codes O13–16), gestational diabetes (ICD-9 codes 648.0 and 648.8 or ICD-10 code O24), placenta previa (ICD-9 codes 641.0 and 641.1 or ICD-10 codes O44), placenta abruption (ICD-9 codes 641.2 and 641.3 or ICD-10 codes O45), preterm premature rupture of membranes (PPROM) (ICD-9 code 666 or ICD-10 codes O72), postpartum hemorrhage (PPH) (ICD-9 code 658 or ICD-10 codes O42). Ovarian hyperstimulation syndrome (OHSS) was also recorded. (3) Neonatal birth outcomes: Sex ratio, Preterm birth, birth weight, classification as small for gestational age (SGA) or large for gestational age (LGA), and cesarean section rate.

### Statistical analysis

The differences among the four groups in baseline characteristics, including maternal age, paternal age, number of embryos, and cause of infertility, were analyzed using the chi-square test. To reduce the effects of confounding that may occur because of potential differences in the distribution of measured baseline characteristics among groups in observational studies. We then employed inverse probability of treatment weighting (IPTW) to balance the baseline characteristics among groups by calculating the propensity score via a multinominal logistic regression analysis. Subsequently, we weighted each group by the inverse of the probability of their treatment allocation and created the pseudo data set (17). A weighted χ2 test and standardized mean difference (SMD) were utilized to assess the balance of baseline characteristics among the groups. An SMD less than 0.1 defined the balance between the groups.

Adjusted multivariate logistic regressions, weighted by IPTW, were conducted to compare pregnancy outcomes, maternal outcomes, and neonatal birth outcomes across the four groups. Maternal age, paternal age, number of embryos transferred, and the cause of infertility were included as adjusted variables. The analysis was performed using SAS 9.4 (SAS Institute Inc., Cary, NC), with a significance level set at P < 0.05 (two-tailed).

## Results

### Characteristics of the study population

The final cohort study encompassed eligible 51762 female participants who had undergone ART and embryo transfer. Prior to the application of Inverse Probability of Treatment Weighting (IPTW), notable disparities were observed between four groups. The blastocyst groups exhibited a higher percentage of women under 35 years old (43.89% in fresh blastocyst vs. 26.92% in fresh cleavage stage; 42.68% in frozen blastocyst vs. 30.65% in frozen cleavage stage). Conversely, the cleavage stage groups had a greater proportion of women aged 40 and above, both in the frozen and fresh embryo transfer groups (13.29% in fresh blastocyst vs. 29.52% in fresh cleavage stage; 13.95% in frozen blastocyst vs. 26.68% in frozen cleavage stage). The same trend was observed for paternal age, with the blastocyst groups having a higher percentage of fathers aged less than 40 years old (72.72% in fresh blastocyst vs. 61.49% in fresh cleavage stage; 72.55% in frozen blastocyst vs. 63.49% in frozen cleavage stage). Conversely, the cleavage stage groups had a greater percentage of fathers aged 40 and above (27.28% in fresh blastocyst vs. 38.51% in fresh cleavage stage; 27.45% in frozen blastocyst vs. 36.51% in frozen cleavage stage). The blastocyst groups showed a higher prevalence of younger mothers and fathers, while the cleavage stage groups exhibited a greater proportion of older individuals. These age-related trends were observed in both fresh and frozen embryo transfer scenarios. Regarding the number of embryos transferred, single embryo transfer (SET) rates were highest in frozen blastocyst transfers (21.26%) and lowest in frozen cleavage transfers (3.61%).

After following the implementation of IPTW, there were no significant disparities observed in factors related to maternal age, paternal age, the number of embryos transferred, or the cause of infertility among the four groups. The distribution of individuals across different groups after IPTW was as follows: 18754 for fresh cleavage stage, 5551 for fresh blastocyst, 9538 for frozen cleavage stage, and 17926 for frozen blastocyst. The Standardized Mean Differences (SMD) for these variables indicated balance after IPTW adjustment (**Supplementary Table 1**).

### Outcome table

Frozen blastocyst transfers exhibited higher rates of biochemical pregnancy (OR 1.34, 95%CI 1.26–1.43), clinical pregnancy (OR 1.33, 95%CI 1.25–1.42), ultrasound confirmation of fetal heartbeats (OR 1.33, 95%CI 1.25–1.42), and live births (OR 1.27, 95%CI 1.19–1.36) when compared to fresh blastocyst transfers. In contrast, frozen cleavage stage transfers had lower rates of biochemical pregnancy (OR 0.80, 95%CI 0.76–0.84), clinical pregnancy (OR 0.73, 95%CI 0.69–0.77), ultrasound confirmation of fetal heartbeats (OR 0.80, 95%CI 0.75–0.84), and live births (OR 0.79, 95%CI 0.74–0.84) compared to fresh cleavage stage transfers (**Table 1**).

**Table 1:** Pregnancy outcomes and adjusted multivariate logistic regression model^a^.

	Fresh Embryo Transfer (N=24,305)				Frozen Embryo Transfer (N=27,464)					Adjusted multivariate logistic regression model^a^			
										Fresh blastocyst versus Fresh cleavage stage	Frozen blastocyst versus Frozen cleavage stage	Frozen cleavage stage versus Fresh cleavage stage	Frozen blastocyst versus Fresh blastocyst
	Fresh cleavage stage (Day2-3) (N=18,754)		Fresh blastocyst (Day 5-6) (N=5,551)		Frozen cleavage stage (Day2-3) (N=9,538)		Frozen blastocyst (Day 5-6) (N=17,926)		p-value				
	N	%	N	%	N	%	N	%					
Biochemical pregnancies (elevated bHCG)	6,925	36.92	2,597	46.78	3,044	31.91	9,632	53.73	<.0001	1.54(1.44-1.63)*	2.59(2.45-2.73)*	0.80(0.76-0.84)*	1.34(1.26-1.43)*
Clinical pregnancies (gestational sac)	6,682	35.63	2,514	45.28	2,753	28.86	9,331	52.05	<.0001	1.53(1.43-1.62)*	2.79(2.64-2.94)*	0.73(0.69-0.77)*	1.33(1.25-1.42)*
Ultrasound confirmation of fetal heartbeat	5,742	30.62	2,090	37.65	2,489	26.09	7,921	44.19	<.0001	1.40(1.31-1.49)*	2.33(2.21-2.47)*	0.80(0.75-0.84)*	1.33(1.25-1.42)*
Live birth	5,072	27.05	1,900	34.24	2,168	22.73	7,085	39.52	<.0001	1.44(1.35-1.54)*	2.32(2.19-2.46)*	0.79(0.74-0.84)*	1.27(1.19-1.36)*

a Adjusted for maternal age, paternal age, number of embryos transferred, and cause of infertility.

*P value<0.05.

FETs were associated with reduced risks of OHSS (OR 0.02, 95%CI 0.02–0.03 for blastocyst and OR 0.05, 95%CI 0.03–0.09 for cleavage stage). However, FETs were linked to a higher risk of pregnancy-induced hypertension (PIH) (OR 1.43, 95%CI 1.19–1.73 for blastocyst and OR 1.23, 95%CI 1.29–1.82 for cleavage stage (**Table 2**). Notably, no significant differences were observed in the incidence of gestational diabetes and preterm premature rupture of membranes (PPROM) among the various transfer groups.For neonatal outcomes, FETs were associated with a higher incidence of LGA infants (OR 1.63, 95% CI 1.30–2.04 for blastocyst and OR 1.69, 95% CI 1.36–2.01 for cleavage stage) and, conversely, a lower incidence of small for gestational age infants (OR 0.68, 95% CI 0.56–0.82 for blastocyst and OR 0.68, 95% CI 0.56–0.82 for cleavage stage). A higher rate of cesarean sections was observed in infants born from frozen embryo transfers (OR 1.66, 95% CI 1.38–1.88 for blastocyst and OR 1.48, 95% CI 1.32–1.67 for cleavage stage). No significant difference was observed in the occurrence of preterm labor or newborn body weight between blastocyst and cleavage stage transfers. The reduced risk of preterm labor in FETs was observed specifically in blastocyst transfer cycles (OR 0.80, 95% CI 0.68–0.95), whereas no such reduction was found in fresh transfer cycles (**Table 3**).

**Table 2:** Maternal outcomes and adjusted multivariate logistic regression model^a^.

	Fresh Embryo Transfer (N=24,478)				Frozen Embryo Transfer (N=28,031)					Adjusted multivariate logistic regression model^a^			
										Fresh blastocyst versus Fresh cleavage stage	Frozen blastocyst versus Frozen cleavage stage	Frozen cleavage stage versus Fresh cleavage stage	Frozen blastocyst versus Fresh blastocyst
	Fresh cleavage stage (Day2-3) (N=18,730)		Fresh blastocyst (Day 5-6) (N=5,673)		Frozen cleavage stage (Day2-3) (N=9,525)		Frozen blastocyst (Day 5-6) (N=18,086)		p-value				
	N	%	N	%	N	%	N	%		OR (95% CI)	OR (95% CI)	OR (95% CI)	OR (95% CI)
Ovarian hyperstimulation syndrome (OHSS)	1,019	5.43	689	12.41	23	0.24	65	0.36	<.0001	2.18(1.89-2.52)*	0.98(0.50-1.91)	0.05(0.03-0.09)*	0.02(0.02-0.03)*
Pregnancy induced hypertension	351	5.06	141	5.43	229	7.53	730	7.58	<.0001	1.09(0.89-1.34)	1.02(0.88-1.20)	1.23(1.29-1.82)*	1.43(1.19-1.73)*
Gestational DM	755	10.90	311	11.99	337	11.08	1,120	11.63	0.3314	1.11(0.96-1.28)	1.06(0.93-1.21)	1.01(0.88-1.16)	0.97(0.85-1.11)
Placenta previa	357	5.15	195	7.50	132	4.35	463	4.81	<.0001	1.49(1.24-1.79)*	1.12(0.92-1.37)	0.83(0.68-1.02)	0.62(0.52-0.74)*
Placenta abruption	361	5.21	173	6.66	242	7.93	713	7.40	<.0001	1.32(1.10-1.60)*	0.94(0.81-1.09)	1.59(1.34-1.88)*	1.13(0.95-1.34)
Preterm premature rupture of membrane (PPROM)	620	8.96	262	10.10	254	8.34	864	8.97	0.1397	1.17(1.00-1.36)*	1.10(0.95-1.28)	0.94(0.81-1.09)	0.89(0.77-1.03)

a Adjusted for maternal age, paternal age, number of embryo transferred, and cause of infertility.

*P value<0.05.

**Table 3:** Neonatal outcomes and adjusted multivariate logistic regression model^a^.

		Fresh Embryo Transfer (N=5,118)				Frozen Embryo Transfer (N=6,631)								
		Fresh cleavage stage (Day2-3) (N=3,763)		Fresh blastocyst (Day 5-6) (N=1,355)		Frozen cleavage stage (Day2-3) (N=1,641)		Frozen blastocyst (Day 5-6) (N=4,990)		p-value	Fresh blastocyst versus Fresh cleavage stage	Frozen blastocyst versus Frozen cleavage stage	Frozen cleavage stage versus Fresh cleavage stage	Frozen blastocyst versus Fresh blastocyst
		N	%	N	%	N	%	N	%		OR (95% CI)	OR (95% CI)	OR (95% CI)	OR (95% CI)
Gestational age											1.10(0.92-1.31)	0.98(0.82-1.16)	0.91(0.76-1.08)	0.80(0.68-0.95)*
**Preterm labor** **(week<37)**	extremely preterm (week<28)	24	0.64	18	1.26	7	0.40	45	0.89	0.0179				
Very preterm (28≦week<32)	58	1.52	19	1.34	14	0.87	58	1.13						
Preterm (32≦week<37)	426	11.23	167	11.94	183	11.08	511	10.03						
week≧37		3,288	86.62	1,195	85.47	1,450	87.67	4,486	87.95					
Newborn body weight														
<2500g		498	13.13	183	13.06	175	10.59	465	9.11	<.0001	1.01(0.84-1.21)	0.86(0.72-1.03)	0.78(0.65-0.94)*	0.67(0.55-0.80)*
2500g-4000g		3,267	86.06	1,197	85.61	1,443	87.26	4,508	88.38					
≧4000g		31	0.82	19	1.34	36	2.17	128	2.50		1.56(0.87-2.77)	1.11(0.76-1.62)	2.64(1.63-4.28)*	1.88(1.15-3.07)*
**Small for gestational age (<10th)**		511	13.46	155	11.10	156	9.46	396	7.76	<.0001	0.82(0.67-0.99)*	0.82(0.67-1.00)	0.68(0.56-0.82)*	0.68(0.56-0.82)*
**Large for gestational age (>90th)**		211	5.56	96	6.87	150	9.07	550	10.78	<.0001	1.22(0.95-1.57)	1.18(0.97-1.42)	1.69(1.36-2.10)*	1.63(1.30-2.04)*
Route of delivery														
Vaginal delivery		2,005	52.83	749	53.55	703	42.48	2,062	40.43	<.0001				
Cesarian section		1,791	47.18	650	46.47	952	57.54	3,039	59.57	0.95(0.84-1.08)	1.07(0.95-1.20)	1.48(1.32-1.67)*	1.66(1.48-1.88)*

a Adjusted for maternal age, paternal age, number of embryos transferred, and cause of infertility.

*P value<0.05.

## Discussion

Previous studies had demonstrated superior outcomes of FET in terms of pregnancies and live births when compared to fresh ET (12–16). The disparities in pregnancy outcomes between FET and fresh ET can be attributed, in part, to the pivotal role played by the uterine environment. During fresh ET, controlled ovarian stimulation often results in a supraphysiologic level of estrogen (E2) within the maternal body (18). This heightened E2 environment may disrupt the synchronization between the endometrium and the embryo, potentially impacting the implantation process. This effect is particularly pronounced in high-responder PCOS populations as shown in the result presented by Chen et al (12). FETs offers the advantage of eliminating the influence of iatrogenic gonadotropin administration and allowing the ovaries to recover from stimulation. This process enables the endometrium, which may have been affected by ovarian stimulation, to shed and regenerate under less intensive endometrial preparation regimens. Consequently, this can create a more favorable uterine environment for embryo implantation in FETs compared to fresh transfers. Conversely, in normal responder populations, the impact of supraphysiologic E2 is comparatively less pronounced (13, 14).

Our study meticulously considered the developmental stage of embryos, acknowledging the shift from the conventional practice of transferring cleavage-stage embryos on day 3 to the contemporary approach of transferring blastocysts on day 5 or 6. This evolution aims to closely mimic the timing of natural implantation, thereby optimizing the synchronization between the endometrium and embryo development. Over the past decade, there has been a progressive increase in the utilization of blastocyst transfer in assisted reproduction cycles (10, 11). In Taiwan, current practice predominantly involves cultivating embryos to the blastocyst stage whenever feasible, thanks to advancements in vitrification techniques (19). Initial data from our study revealed a higher proportion of older patients opting for cleavage-stage embryo transfers, suggesting a potential lower quality of cleavage-stage embryos in this demographic. It’s important to note that utilizing these potentially more fragile cleavage-stage embryos in FET, compounded by the stress of cryopreservation, may have contributed to less favorable pregnancy outcomes. Available studies indicate that freezing procedures can impact the embryonic cytoskeleton, DNA integrity, and miRNA transcriptome, potentially leading to chromosomal aberrations and imprinting disorders (20–22). An *in vitro* study suggested that vitrification might decrease the viability of mouse embryos through chromosomal aberrations-mediated cell death (22). However, previous research has shown that while embryo cryopreservation can affect embryo quality, it does not necessarily impair the implantation or pregnancy potential of high-quality embryos (23). Cleavage-stage embryos appear to be more sensitive to the stresses associated with cryopreservation compared to blastocyst-stage embryos, resulting in compromised viability and lower success rates following thawing.

The incidence of OHSS is significantly higher in fresh ET cycles compared to FET cycles. This observation aligns with previous research, including Cochrane reviews, which consistently advocate for the freeze-all approach due to its reduced OHSS risk (14, 16, 24, 25). The conclusion that FET is associated with a higher incidence of PIH and LGA also aligns with previous study findings (9, 12, 16, 24, 26, 27). However, the precise mechanisms for the increased risk of PIH and LGA with FET remain unclear. Some research has explored this phenomenon from an epigenetic perspective, suggesting that the external freezing environment may influence processes like miRNA downregulation and DNA methylation, leading to epigenetic dysregulation that affects both placental and fetal growth (16, 26, 28, 29). Such epigenetic dysregulation, in turn, has been associated with abnormal placentation and fetal growth.

Our findings are consistent with prior research indicating that transferring frozen-thawed embryos is linked to a reduced risk of SGA and low birth weight infants compared to fresh embryo transfers (18, 30, 31). Similarly, both our study and previous research have noted a trend towards fewer occurrences of SGA babies following blastocyst transfers (18, 32, 33). However, the issue of preterm labor remains contentious. Our study did not observe any distinction of preterm birth between blastocyst and cleavage stage transfers, which contrasts with prior research reporting significantly higher incidences of preterm birth after blastocyst stage compared to cleavage-stage embryo transfer in fresh cycles (34, 35). Our data suggests a tendency towards a higher risk of preterm labor in fresh embryo transfers, with a notable difference observed in blastocyst but not cleavage stage transfer cycles. The varying risks of preterm birth in fresh and frozen embryo transfer cycles might be potentially due to issues with synchronization between the endometrium and embryos. This timing discrepancy may be more accurately addressed in frozen cycles, impacting the timing of delivery. Furthermore, the hormonal conditions in fresh cycles and inflammatory effects from continuous ovarian stimulation could influence early conception and peri-implantation function (34).

### Strength and limitations

In our study, we leveraged the strength of utilizing national registry data, which is extensive and representative of the entire country, providing us with a comprehensive and authentic dataset from Taiwan. However, it’s important to acknowledge certain limitations. Firstly, the specific endometrial preparation methods, whether hormonal (HRT) or natural cycle, were not available from the reproductive database. Secondly, the absence of certain data points in the reproductive database, such as ovarian reserve, number of oocytes retrieved, and embryo quality may possibly cause bias. Thirdly, our study design, being retrospective, may not offer the same level of rigor as a RCT. Fourthly, due to regulatory constraints in Taiwan, a higher proportion of couples opt for double embryo transfer instead of single embryo transfer after the age of 36. Rather than presenting the reproductive results of single embryo transfers (SET) across the four groups, to address potential enrollment biases resulting from this practice, we have chosen to employ IPTW matching to control for the number of embryo transfers in different groups, thereby mitigating differences in embryo transfer numbers across groups.

## Conclusion

In summary, our study suggests that frozen embryo transfer yields better CPR and LBR when frozen blastocysts are transferred, but these rates are lower when frozen cleavage stage embryos are utilized. Therefore, the freeze-all strategy may not be suitable for universal application. When embryos can develop to the blastocyst stage, especially in cases of better embryo quality and a higher number of embryos, FET is a favorable choice, but caution should be exercised due to a higher risk of PIH, placental abruption, and heavier newborn birthweights. Conversely, lower CRP and LBR in frozen cleavage stage embryo transfers was found according to our data. When embryos can only develop to the cleavage stage, fresh embryo transfer becomes a more reasonable option, but it is associated with a higher risk of OHSS and low birth weight. This underscores the importance of considering embryo developmental stage and cryopreservation techniques in optimizing outcomes in assisted reproduction.

The decision regarding which embryo transfer strategy to employ in the field of assisted reproductive technology is shaped by a multifaceted interplay of factors. These factors encompass the patient’s ovarian response, the stage of embryo development utilized, the quality of embryos, and the potential influence of the supraphysiologic estrogen environment associated with fresh embryo transfer. The diversity in outcomes highlights the necessity for continuous research to enhance and fine-tune the best practices in assisted reproductive technology.

## Data availability statement

The dataset is confined to data sourced from the national assisted reproductive technology database and the National Health Insurance Database. Restrictions may apply to accessing personal or identifiable information to safeguard patient confidentiality and adhere to privacy regulations. Requests to access these datasets should be directed to https://www.hpa.gov.tw/Pages/List.aspx?nodeid=60.

## Ethics statement

The studies involving humans were approved by Kaohsiung Medical University Chung-Ho Memorial Hospital, IRB-No. KMUHIRB-E(I)-20210222. The studies were conducted in accordance with the local legislation and institutional requirements. The ethics committee/institutional review board waived the requirement of written informed consent for participation from the participants or the participants’ legal guardians/next of kin because this is a retrospective cohort study.

## Author contributions

C-TC: Writing – original draft, Writing – review & editing, Conceptualization. S-FW: Data curation, Formal analysis, Writing – review & editing. H-YC: Writing – review & editing. I-LH: Writing – review & editing. C-YH: Writing – review & editing. E-MT: Conceptualization, Supervision, Writing – review & editing.
