# Supplementary material for: Embryo transfer impact: a comprehensive national cohort analysis comparing maternal and neonatal outcomes across varied embryo stages in fresh and frozen transfers
Source: Front Endocrinol (Lausanne). 2024 Jun 12;15:1400255. doi: 10.3389/fendo.2024.1400255 (PMC11199782; doi:10.3389/fendo.2024.1400255)
Supplement: Supplementary file 1 [file Presentation_1.pptx]

## Slide 1
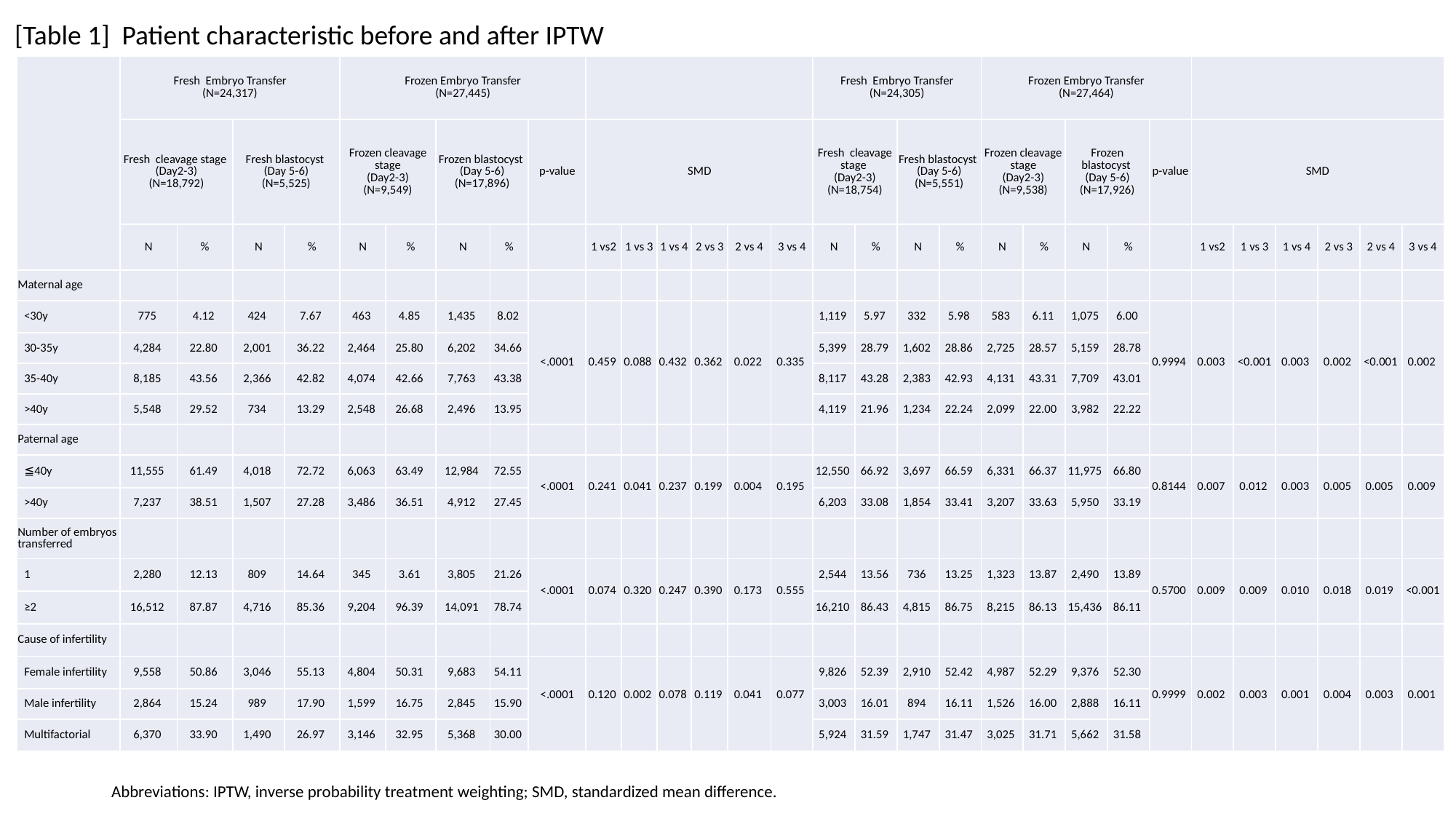

[Table 1] Patient characteristic before and after IPTW
| | Fresh Embryo Transfer (N=24,317) | | | | Frozen Embryo Transfer (N=27,445) | | | | | | | | | | | Fresh Embryo Transfer (N=24,305) | | | | Frozen Embryo Transfer (N=27,464) | | | | | | | | | | |
| --- | --- | --- | --- | --- | --- | --- | --- | --- | --- | --- | --- | --- | --- | --- | --- | --- | --- | --- | --- | --- | --- | --- | --- | --- | --- | --- | --- | --- | --- | --- |
| | Fresh cleavage stage (Day2-3) (N=18,792) | | Fresh blastocyst (Day 5-6) (N=5,525) | | Frozen cleavage stage (Day2-3) (N=9,549) | | Frozen blastocyst (Day 5-6) (N=17,896) | | p-value | SMD | | | | | | Fresh cleavage stage (Day2-3) (N=18,754) | | Fresh blastocyst (Day 5-6) (N=5,551) | | Frozen cleavage stage (Day2-3) (N=9,538) | | Frozen blastocyst (Day 5-6) (N=17,926) | | p-value | SMD | | | | | |
| | N | % | N | % | N | % | N | % | | 1 vs2 | 1 vs 3 | 1 vs 4 | 2 vs 3 | 2 vs 4 | 3 vs 4 | N | % | N | % | N | % | N | % | | 1 vs2 | 1 vs 3 | 1 vs 4 | 2 vs 3 | 2 vs 4 | 3 vs 4 |
| Maternal age | | | | | | | | | | | | | | | | | | | | | | | | | | | | | | |
| <30y | 775 | 4.12 | 424 | 7.67 | 463 | 4.85 | 1,435 | 8.02 | <.0001 | 0.459 | 0.088 | 0.432 | 0.362 | 0.022 | 0.335 | 1,119 | 5.97 | 332 | 5.98 | 583 | 6.11 | 1,075 | 6.00 | 0.9994 | 0.003 | <0.001 | 0.003 | 0.002 | <0.001 | 0.002 |
| 30-35y | 4,284 | 22.80 | 2,001 | 36.22 | 2,464 | 25.80 | 6,202 | 34.66 | | | | | | | | 5,399 | 28.79 | 1,602 | 28.86 | 2,725 | 28.57 | 5,159 | 28.78 | | | | | | | |
| 35-40y | 8,185 | 43.56 | 2,366 | 42.82 | 4,074 | 42.66 | 7,763 | 43.38 | | | | | | | | 8,117 | 43.28 | 2,383 | 42.93 | 4,131 | 43.31 | 7,709 | 43.01 | | | | | | | |
| >40y | 5,548 | 29.52 | 734 | 13.29 | 2,548 | 26.68 | 2,496 | 13.95 | | | | | | | | 4,119 | 21.96 | 1,234 | 22.24 | 2,099 | 22.00 | 3,982 | 22.22 | | | | | | | |
| Paternal age | | | | | | | | | | | | | | | | | | | | | | | | | | | | | | |
| ≦40y | 11,555 | 61.49 | 4,018 | 72.72 | 6,063 | 63.49 | 12,984 | 72.55 | <.0001 | 0.241 | 0.041 | 0.237 | 0.199 | 0.004 | 0.195 | 12,550 | 66.92 | 3,697 | 66.59 | 6,331 | 66.37 | 11,975 | 66.80 | 0.8144 | 0.007 | 0.012 | 0.003 | 0.005 | 0.005 | 0.009 |
| >40y | 7,237 | 38.51 | 1,507 | 27.28 | 3,486 | 36.51 | 4,912 | 27.45 | | | | | | | | 6,203 | 33.08 | 1,854 | 33.41 | 3,207 | 33.63 | 5,950 | 33.19 | | | | | | | |
| Number of embryos transferred | | | | | | | | | | | | | | | | | | | | | | | | | | | | | | |
| 1 | 2,280 | 12.13 | 809 | 14.64 | 345 | 3.61 | 3,805 | 21.26 | <.0001 | 0.074 | 0.320 | 0.247 | 0.390 | 0.173 | 0.555 | 2,544 | 13.56 | 736 | 13.25 | 1,323 | 13.87 | 2,490 | 13.89 | 0.5700 | 0.009 | 0.009 | 0.010 | 0.018 | 0.019 | <0.001 |
| ≥2 | 16,512 | 87.87 | 4,716 | 85.36 | 9,204 | 96.39 | 14,091 | 78.74 | | | | | | | | 16,210 | 86.43 | 4,815 | 86.75 | 8,215 | 86.13 | 15,436 | 86.11 | | | | | | | |
| Cause of infertility | | | | | | | | | | | | | | | | | | | | | | | | | | | | | | |
| Female infertility | 9,558 | 50.86 | 3,046 | 55.13 | 4,804 | 50.31 | 9,683 | 54.11 | <.0001 | 0.120 | 0.002 | 0.078 | 0.119 | 0.041 | 0.077 | 9,826 | 52.39 | 2,910 | 52.42 | 4,987 | 52.29 | 9,376 | 52.30 | 0.9999 | 0.002 | 0.003 | 0.001 | 0.004 | 0.003 | 0.001 |
| Male infertility | 2,864 | 15.24 | 989 | 17.90 | 1,599 | 16.75 | 2,845 | 15.90 | | | | | | | | 3,003 | 16.01 | 894 | 16.11 | 1,526 | 16.00 | 2,888 | 16.11 | | | | | | | |
| Multifactorial | 6,370 | 33.90 | 1,490 | 26.97 | 3,146 | 32.95 | 5,368 | 30.00 | | | | | | | | 5,924 | 31.59 | 1,747 | 31.47 | 3,025 | 31.71 | 5,662 | 31.58 | | | | | | | |
Abbreviations: IPTW, inverse probability treatment weighting; SMD, standardized mean difference.
